# Swallowing and feeding of young children on high-flow oxygen therapy

**DOI:** 10.4102/sajcd.v71i1.1010

**Published:** 2024-03-02

**Authors:** Ruhee Hoosain, Bhavani Pillay, Shabnam Abdoola, Marien A. Graham, Esedra Krüger

**Affiliations:** 1 Department of Speech-Language, Pathology and Audiology, Faculty of Humanities, University of Pretoria, Pretoria, South Africa; 2 Department of Science, Mathematics and Technology Education, Faculty of Education, University of Pretoria, Pretoria, South Africa

**Keywords:** swallowing, feeding, high-flow oxygen, oral motor characteristics, SOMA, speech-language therapist, burns

## Abstract

**Background:**

Oral feeding practices of young patients on high-flow oxygen (HFO_2_) have been controversial. Limited literature exists on this topic, but new studies suggest introducing oral feeds.

**Objective:**

This study aims to describe the changes in swallowing and feeding of a group of young children on HFO_2_.

**Method:**

Twelve participants (mean age 34.17 months [s.d. = 3.97]) on HFO_2_ were assessed clinically at the bedside using the Schedule of Oral Motor Assessment. Assessments were conducted twice to determine the change in characteristics: upon approval from the managing doctor when respiratory stability on HFO_2_ was achieved and for a second time on the last day of receiving HFO_2_ (mean 2.6 days apart). Patients received standard in-patient care and speech therapy intervention.

**Results:**

Most participants displayed typical oral motor function at initial and final assessments for liquid, puree and semi-solid consistencies. Purees and soft solid consistencies were introduced to most participants (*n* = 11, 91.7%). Solids and chewables were challenging for all participants during both assessments. Half of the participants displayed gagging and a wet vocal quality with thin liquids at the initial assessment only.

**Conclusion:**

This small-scale study found that HFO_2_ should not preclude oral diets, but in this sample, small amounts of oral feeding could be introduced with caution, in an individualised manner, and with a collaborative multidisciplinary approach. Further research is essential.

**Contribution:**

Partial oral feeding of specific consistencies was possible during the assessment of young paediatric in-patients on HFO_2_. Monitoring of individual patient characteristics and risk factors by a specialist feeding team is essential.

## Introduction

Respiratory distress in children is often treated using non-invasive ventilation such as high-flow oxygen (HFO_2_) therapy via nasal cannula (Singer & Rattanachaiwong, 2018). The term high-flow oxygen can be used interchangeably with high-flow nasal cannula (HFNC) oxygen therapy: a non-invasive ventilation method where a humidified combination of oxygen and air is delivered through binasal tubes at gas flow rates of more than 1 litre per minute (Charlton et al., 2022). Published literature on the safety of oral feeding in patients on HFO_2_ is inconsistent (Barnes et al., 2023; Murphy et al., 2018). Non-invasive ventilation in children increases the diameter of the airway (Murphy et al., 2018). Consequently, there is concern that oral feeding in patients on HFO_2_ may cause swallowing-related respiratory decline (Maitland et al., 2018; Murphy et al., 2018; Singer & Rattanachaiwong, 2018). There is substantial evidence advocating for early enteral nutrition in patients on non-invasive ventilation (Murphy et al., 2018); however, oral feeding during HFO_2_ is not yet common practice.

A paucity of research with conflicting conclusions regarding feeding on any form of ventilator support has left speech-language therapists (SLTs) and other health professionals without clear evidence to guide feeding decisions for young children on HFO_2_ (Murphy et al., 2018). The role of SLTs in paediatric critical care settings, where most patients are on ventilator support, is predominantly in the assessment and management of swallowing and feeding (Murphy et al., 2018; Singer & Rattanachaiwong, 2018). The disadvantage of the initiation of oral feeds while on non-invasive ventilation is that it may induce respiratory distress (Capilouto, 2017). In patients with respiratory distress, reduced energy or possible neurological difficulty could result in oropharyngeal dysphagia (OPD) and possible aspiration (Capilouto, 2017).

Oral intake abstention resulting from HFO_2_ increases the possibility of oral sensorimotor systems being affected, thus impacting later feeding (Capilouto, 2017). Food refusal and fussy eating may stem from feeding and swallowing dysfunction, unpleasant oral experiences while on non-invasive ventilation, or long-term tube feeding resulting in under usage of oral-motor structures (Capilouto, 2017). A recent stance is that HFO_2_ should not preclude oral diets, but oral intake should be practised with caution when a child is stable on HFO_2_ (Singer & Rattanachaiwong, 2018).

Respiratory stability on HFO_2_ is usually achieved after the flow rate is determined and monitored by a senior physician (Maitland et al., 2018). The weight of the child helps to determine the starting flow rate to achieve respiratory stability on HFO_2_ (10 kg: 2 L/kg/min [Litres/kilogram/minute] for the first 10 kg plus 0.5 L/kg/min for each kg above 10 kg, with a maximum flow rate of 50 L/min) (Maitland et al., 2018). However, there are no known guidelines in South Africa for SLTs to guide feeding decisions when young children are on HFO_2_.

There are varying perceptions and practices among members of the multidisciplinary team for feeding ventilator-dependent paediatric in-patients (Barnes et al., 2023). Current published data are predominantly originating in high-income countries with access to HFO_2_ at healthcare facilities (McCall, 2019; Murphy et al., 2018). South Africa is considered an upper-middle-income country, with a high proportion of people with a low socio-economic status (Sochet et al., 2017).

This under-resourced population is faced with risks such as poor living conditions, and children have an increased number of comorbidities, including lower respiratory tract infections, for which HFO_2_ may be warranted (Sochet et al., 2017). However, demographically, diagnoses may also be attributed to a lower socio-economic status itself, as people often live and work in environments that have a higher risk for pollution, psychosocial stressors, trauma and injury, and vulnerability to disease with poor outcomes (Marutlulle, 2021).

## Research methods and design

### Aim and research design

The aim of this study was to describe swallowing and feeding characteristics in a group of young in-patient children on HFO_2_. This exploratory study set out to describe a preliminary profile of swallowing and feeding characteristics in a small group of young children on HFO_2_. It consisted of a prospective longitudinal study design with repeated measures at two points to track the change in swallowing and feeding characteristics over time.

### Setting

The research site was a large tertiary hospital in South Africa. Participants were from paediatric units which cater for paediatric inpatients who require HFO_2_ i.e., the paediatric high care unit, paediatric burns unit and intensive care unit. Often, patients in these wards are on HFO_2_ for various diagnoses such as burn and trauma injuries, chronic lung disease, bronchiolitis, lower respiratory tract infections and severe acute malnourishment. These units accommodated parents only throughout the day’s visitation times, where parents often assisted with feeding children if they were not on alternative methods of feeding such as oro-gastric or naso-gastric tubes.

### Participants

Twelve participants who met the inclusion criteria (Table 1) were prospectively included using snowball sampling (Table 2) as they were referred for assessments from the medical doctor.

**TABLE 1:** Participants’ inclusion and exclusion criteria.

Inclusion criteria	Exclusion criteria
Within the age range of 2–4 years.	Physically too ill to feed orally, therefore, exclusively on alternate feeds (oro-gastric or naso-gastric feeding tubes).
Admitted into a paediatric ward on HFO_2_, either as initially required or following respiratory instability on nasal prongs oxygen therapy.	Patients who are on nasal prongs oxygen therapy with a flow rate of >40 L/min as there is a significantly higher risk of aspiration or respiratory decline (Sharma et al., 2021).
HFO_2_ set at a flow rate of ≤ 40 L/min as higher flow rates are said to have potential risks of aspiration and difficulties swallowing (Sharma et al., 2021).	Co-existing neurological conditions such as cerebral palsy.
Glascow Coma Scale (GCS) score of 9 to 15 out of 15 (Jain & Iverson, 2019).	Symptoms of or under investigation for COVID-19.
Medically stable for at least one day before SLT feeding and swallowing assessment as determined by the doctor and file review.	Caregiver displaying symptoms of or under investigation for COVID-19.
Already has an established oral feeding method prior to hospital admission. This was so that the SLT can commence assessment from the participants’ known feeding method.	-
Must be on an alternate mode of feeding (oro-gastric or naso-gastric feeding tubes) while on HFO_2_ and ready to transition to oral feeding.	-
Parent or legally appointed caregiver, 18 years or older for legally providing consent.	-
Consent from the physician that the patient can be fed orally.	-

Note: Please see the full reference list of the article, Hoosain, R., Pillay, B., Abdoola, S., Graham, M.A., & Krüger, E. (2024). Swallowing and feeding of young children on high-flow oxygen therapy. *South African Journal of Communication Disorders*, 71(1), a1010. https://doi.org/10.4102/sajcd.v71i1.1010, for more information.

HFO_2_, high-flow oxygen; L/min, litres/minute; SLT, speech-language therapist; COVID-19, coronavirus disease 2019.

**TABLE 2:** Participants’ description (*N* = 12).

Participants’ characteristics	Mean	s.d.	*n*	%
Age (month)	34.17	3.97	-	-
Gestational age (weeks)	38	1	-	-
Birth weight in kg	2.67	0.43	-	-
Current weight in kg	14.27	2.96	-	-
Male	-	-	8	66.7
Female	-	-	4	33.3
**Home description:**	-	-		
Shack	-	-	4	33.3
Room	-	-	1	8.3
House	-	-	7	58.3
Access to clean water	-	-	12	100
Access to electricity	-	-	12	100
Access to a toilet	-	-	12	100
Prematurity	-	-	1	8.3
Epilepsy and seizures × 2 shortly after birth but no neurological-related deficits as a result	-	-	1	8.3
**Reasons for hospital admission**				
Minimum of 20% and maximum of 40% TBSA hot water burns†	-	-	6	50
30% TBSA flame burns‡	-	-	1	8.3
Bronchopneumonia	-	-	3	25
Gunshot wound	-	-	1	8.3
Near-drowning experience§	-	-	1	8.3

† , including the head, face and neck regions;

‡ , including the face, neck and intra-oral regions;

§ , less than 5 min with no neurological fallout as determined by the assessing physician and recorded in the patient file.

kg, kilograms; TBSA, total body surface area; s.d., standard deviation.

### Procedure

Data collection took place between June 2021 and December 2021. Participants were seen post-admission after the medical file review, discussion with the managing doctor and when potential participants met inclusion criteria. Participants’ caregivers were asked questions in a private space or telephonically. The researcher was adequately qualified and trained in performing the clinical swallowing and feeding assessment and collected data independently. A second rater assessed 15% of the sample. The second rater was a qualified SLT for 4 years, not involved with the study, but trained in the procedures. Inter-rater agreement was obtained at a kappa of 0.981 indicating almost perfect agreement increasing the reliability of the findings.

Each participant was initially assessed at their bedside once respiratory stability was established on HFO_2_ and once again on the day when participants concluded the HFO_2_ therapy (i.e. a follow-up assessment, to track changes in swallowing and feeding characteristics). The two assessments were conducted a mean of 2.6 days apart for the participants in this study. The observation of swallowing was ensured to identify visible signs of aspiration such as a maintained drop in the participant’s SPO_2_, coughing, wet vocal quality, intercostal recessions, or difficulty breathing, colour of extremities changing blue as well as a rise in temperature accompanied by sweating (Tosif & Duke, 2017). Verbal feedback was provided to caregivers following assessments. Participants with difficulties were referred to medical and allied healthcare professionals for treatment.

The two assessments were conducted across four food consistencies (liquids, pureed, semi-solid and solid) using the Schedule for Oral Motor Assessment (SOMA) (Reilly et al., 1995). The SOMA was conducted as part of a bedside swallowing and feeding assessment at two points in time, and observations of swallowing were also made. Attempts were made to feed all participants as follows: by cup for liquid consistencies, by spoon for semi-solid and solid consistencies, and independently for orally consuming a finger food (e.g. a biscuit) (Table 3).

**TABLE 3:** Food adaptations for the Schedule for Oral Motor Assessment used in this study.

Food type	SOMA-suggested-food	Food adaptations for the current study	IDDSI description
1. Liquids	Any safe liquids for drinking	Milk, water or juice provided by the hospital at feeding times	Thin IDDSI level 0
2. Puree	Chocolate mousse Fromage frais or thick yoghurt Pureed fruit	Thick yoghurt	Mildly thick IDDSI level 2–3 and 4
3. Semi-solid	Plain cottage cheese Petit pois†	Soft porridge or mashed banana	Moderately thick IDDSI level 5
4. Solid	Potato salad Mixed fruit‡	Cooked oats or dried fruit	Minced and moist IDDSI level 6
5. Chewable biscuits	Crisp-bread or cheese snack biscuits Savoury crackers or digestive biscuits Oat cakes or ginger-nut biscuits	Soft chew – Marie biscuit Medium chew – Good Morning biscuits Hard chew – Nutticrust	Easy to chew IDDSI level 7

† , baby peas, warm or cold according to preference;

‡ , apple or apricot or pear.

IDDSI, International Dysphagia Diet Standardisation Initiative; SOMA, Schedule for Oral Motor Assessment.

Each consistency used in the SOMA was adapted to suit the local context, that is, food that was affordable and known to participants, as in a previous study (Fuls et al., 2020). The International Dysphagia Diet Standardisation Initiative (IDDSI) was used to describe the textures of food and the thickness of liquids used in the assessments of the participants using the SOMA (Cichero et al., 2017). International Dysphagia Diet Standardisation Initiative has established consistency assessments ranging from using a syringe to a spoon or fork in testing a consistency in question to determine where on the continuum of thickness does it lie (scale of 0 [thin consistencies] to 7 [food with no texture restrictions, i.e., hard textures requiring biting and chewing]) (Cichero et al., 2017).

### Ethical considerations

Institutional ethical clearance was obtained before data collection commenced at the University of Pretoria, Faculty of Health Sciences, #HUM012/1220.

## Results and discussion

Preliminary information about oral motor function when being introduced to oral feeding on HFO_2_ was obtained in this small exploratory study of 12 children. Signs of OPD were identified. The two repeated assessments showed changes in participants’ ability to be assessed with a wider variety of consistencies on the SOMA. Participants were respiratory stable on HFO_2_ at a mean level of 19.17 L/kg/min (s.d. = 4.82 L/kg/min) at the initial assessment (Table 4). More than half of the participants managed oral intake of various consistencies at both the initial and final swallowing and feeding assessments within what is expected of their developmental skills at their age (Table 5). Even though participants were medically stable according to inclusion criteria, participants were still displaying behaviours of being weak or lethargic and listless. Therefore, because of the energy required to self-feed and chew the trials of chewable foods, some participants could not be assessed with this owing to visibly presenting poorly or refusing the trial. Subsequently, prior to the final assessment, HFO_2_ was weaned to a mean level of 10.73 L/kg/min (s.d. = 4.96 L/kg/min) because of participants slowly becoming respiratory stable and coping on lower levels (Table 4). There were two participants who had a decline in their respiratory state requiring them to be re-intubated. These patients could not be assessed because of a decline in their medical stability. These findings are consistent with those of Canning et al. (2021) who concluded that it was not the use of HFO_2,_ but rather patient-specific factors of swallowing and feeding readiness and underlying medical conditions that affected readiness for oral intake.

**TABLE 4:** The high-flow oxygen and oxygen saturation level levels at the initial and final assessments (mean 2.6 days apart).

L/kg/min and SPO_2_	Mean	s.d.
**Initial assessment**		
Level of HFO_2_ at the initial assessment L/kg/min	19.17	4.82
Initial SPO_2_ (%)	98.5	1.16
**Final assessment**		
Level of HFO_2_ at the final assessment L/kg/min	10.7	4.96
Final SPO_2_ (%)	99.4	0.68

HFO_2_, high-flow oxygen; s.d., standard deviation; SPO_2_, Percentage of oxygen saturation; L/kg/min, litres/kilogram/minute.

**TABLE 5:** Initial and final Schedule for Oral Motor Assessment results (*N* = 12).

Characteristics†	Initial assessment		Final assessment	
	*n*	%	*n*	%
**Liquids**				
Oral motor dysfunction	0	-	0	-
Normal oral motor function	12	100	11	91.7
CNT because of patient state	-	-	1	8.3
Signs of penetration and/or aspiration	4	33.3	1	8.3
**Puree**				
Oral motor dysfunction	0	-	0	-
Normal oral motor function	11	91.7	11	91.7
CNT because of patient medical stability	1	8.3	1	8.3
**Semi-solids**				
Oral motor dysfunction	0	-	0	-
Normal oral motor function	11	91.7	11	91.7
Refused	1	8.3	0	-
CNT because of patient medical stability	-	-	1	8.3
**Solids**				
Oral motor dysfunction	0	-	0	-
Normal oral motor function	7	58.3	10	83.3
CNT because of patient medical stability	5	41.7	2	16.7
**Biscuits**				
**Soft chew**				
Oral motor dysfunction	0	-	0	-
Normal oral motor function	6	50	10	83.3
CNT because of patient medical stability	6	50	2	16.7
**Medium chew**				
CNT because of patient medical stability	12	0	12	0
**Hard chew**				
CNT because of patient medical stability	12	0	12	0

Note: Medium chew and hard chew final assessment *N* = 0.

† , consistencies assessed.

CNT, could not test.

Previously, there was controversy in feeding children orally on HFO_2_ and the hesitancy is likely derived from concerns regarding aspiration risk (Murphy et al., 2018). A recent systematic review’s findings on oral feeding while on HFO_2_ were insufficient to provide a concluding stance (Canning et al., 2021). Thus, to date, the effects of HFO_2_ on swallowing and feeding are still not well elucidated, which results in varied feeding practices and intervention recommendations provided by SLTs (Rice & Lefton-Greif, 2022). This small-scale study found that HFO_2_ should not preclude oral diets, but in this sample, small amounts of oral feeding could be introduced with caution, in an individualised manner, while collaborating closely with the multidisciplinary team involved. Further research would be beneficial. Instrumental assessment would be of value to determine the presence of silent aspiration.

Prior to the initial assessment, the treating physician provided participants with medical clearance to partake in oral feeding. Participants were found to be awake, alert, and responsive and were repositioned into a semi-reclined position for the assessment. All participants had GCS between 12 and 15 at both assessments. The findings show that those participants who could be assessed (*n* = 10), displayed mostly normal oral-motor function for liquids, pureed and semi-solid consistencies at both initial and final assessments of swallowing and feeding (Table 5). The volumes of trials offered varied as tolerated by participants. Feeding difficulties that were identified in this sample were: refusal of certain consistencies requiring increased effort to swallow, or the participant’s medical state contributing to reduced or no oral intake, and an increased duration of feeding. Liquid consistencies (IDDSI level 0) may have contributed to a possible aspiration event when the amount of high flow oxygen was increased (mean 19.17 L/kg/min; s.d. = 4.82 L/kg/min) as coughing was observed. This would have to be confirmed using a larger sample. While the feasibility of instrumental assessment remains an issue in a country such as South Africa where videofluoroscopic swallow studies are not readily available (Coutts & Pillay, 2021), it will be valuable to conduct future research with similar populations using instrumental assessments to determine the presence of silent aspiration.

In this study, the liquid consistency put participants at a possible risk of aspiration at the initial assessments (*n* = 4, 33.3%). The liquid consistency was safely administered at the final assessment while still on HFO_2_. Pureed, semi-solid and solid consistencies were safely administered when respiratory stability was achieved following the initiation of HFO_2_. This also proved to be the case when HFO_2_ was being weaned because of slowly achieving respiratory stability in those participants who were medically and physically stable to feed orally. The less viscous the liquid consistency, the higher the chances of an aspiration event without oxygen therapy (Wolter et al., 2018). Therefore, with oxygen therapy and a higher flow rate, thin liquids may be more difficult to control while swallowing (Steele et al., 2015).

Purees and semi-solids were tolerated well in all participants at both assessments. Liquids and soft chew consistencies were only possible at the second attempt. The SLTs could therefore, consider a careful approach to liquid trials with young patients on HFO_2_ in future research endeavours. Evidence suggests that thicker consistency trials such as IDDSI level 2 prior to thin liquid trials may be a consideration to reduce possible penetration and/or aspiration events, however, post-swallow pharyngeal residue should ideally be monitored using instrumental means (Steele et al., 2015), which is not always an option at hospitals in South Africa. In the study, participants who orally consumed thicker consistencies and solids (IDDSI levels 2–6) appeared to have swallowed successfully and did not display observable signs of oral residue post-swallow. Solids and chewable consistencies (IDDSI level 7) were not introduced successfully in this study, often because of participants’ medical state.

**FIGURE 1:**
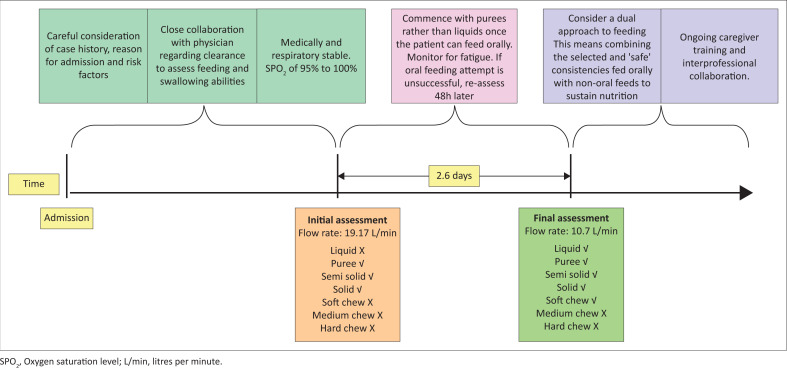
Proposed approach to oral feeding assessment and subsequent management of young children on HFO_2_ based on preliminary study finding.

While this study’s findings are based on a small sample, the value of the findings is that it paves the way for future research. A two-point measurement showed change in the swallowing and feeding ability of participants. Generalisability of findings will not be possible, but a preliminary oral feeding framework was conceptualised based on the observations from this small-scale local study (Figure 1). Participants may have coped on smaller amounts of soft food because of their medical stability and overall state. Clinically, this could be suggestive of ‘partial oral feeds’ on HFO_2_, that is, orally feeding consistencies that the individual coped on at the assessment supplemented by enteral feeds as a means of maximum nutritional intake (Wolter et al., 2018). In this study, participants made progress from the first assessment to the second assessment evidenced by the number of participants who were able to take liquid and soft chew consistencies at the second attempt. Oral feeding ability should thus be specified for the consistency and the amount used. Close consultation with a medical doctor and dietician is necessary. Larger prospective studies are also warranted in this regard. It is important that the multidisciplinary feeding team considers patients holistically and identifies oral feeding as a common goal while working together to ensure nutrition is provided safely (Rice & Lefton-Greif, 2022).

The prominent environmental risk in this study was being born into families with a low socio-economic status from informal settlements. Most participants in this study had trauma-related burns to the body (maximum TBSA of 40%) because of hot water burns. Furthermore, a history of bronchopneumonia proved to be another frequent reason for hospital admission in this study. Such diagnoses may be attributed to a lower socio-economic status as people often live and work in environments that have higher daily living risk factors (Marutlulle, 2021). The observations in this study showed participants on HFO_2_ who commenced oral trials successfully were weaned from HFO_2_ quicker than participants who did not, but well-designed comparative studies are necessary to confirm this finding. The commencement of oral feeding while on HFO_2_ is slowly being supported as it could facilitate transition to full oral feeding without adverse events (Singer & Rattanachaiwong, 2018). Findings in the current exploratory study are insufficient to conclude this stance. The value of the findings are the age of participants studied (mean = 34.17 months) for which there is limited published data, although the sample was small. Larger prospective studies will be valuable.

Considering the role of SLTs within the paediatric intensive care unit, where professionals are faced with assessments for oral feeding readiness with young children on HFO_2_ as the middle ground between either intubation or nasal prongs or room air. The study provides preliminary findings on which further research may be based. SLTs may attempt oral feeding assessment using the puree, semi-solid or solid with young children cautiously, but more confidently than before. However, careful monitoring and individualised decisions remain important. Findings are useful, but further research is required to support SLTs in making complex feeding decisions for high-risk children on HFO_2_.

Studies employing instrumental assessments such as videofluoroscopy or flexible endoscopic evaluation of swallowing could be of value but are not readily available to South African SLTs. SLTs and feeding teams must proceed cautiously, considering the complexity of oral feeding in patients with respiratory distress. Team-based decisions and inter-professional collaboration remain crucial for all healthcare professionals working with swallowing and feeding in vulnerable populations.

## Conclusion

This study’s findings are not generalisable, but is the starting point for further prospective research on the paediatric population requiring HFO_2_. Repeated measures in this longitudinal study showed changes in the participants’ ability to tolerate liquid and soft chew consistencies. Findings highlighted patient-specific determinants such as the medical diagnosis, reason for admission or state of alertness, often lead to respiratory compromise affecting patients’ readiness for different consistencies of oral alimentation. Prospectively collected data were obtained providing valuable information, which is supportive for future research efforts. The findings may be valuable to all healthcare providers such as SLTs working with children on HFO_2_ within similar settings.
